# Introducing the Journal of Pathology Informatics

**DOI:** 10.4103/2153-3539.63821

**Published:** 2010-05-26

**Authors:** Liron Pantanowitz, Anil V. Parwani

**Affiliations:** Department of Pathology, University of Pittsburgh Medical Center, Pittsburgh PA, USA

We are pleased to introduce the **Journal of Pathology Informatics**(JPI), the official journal of the *Association for Pathology Informatics* (API). [1] JPI is an open-access, peer-reviewed journal dedicated to the advancement of Pathology Informatics. Prior to 1980, informatics was more widely practiced in Pathology than in general medicine. [2]The American Board of Pathology actively pursued the establishment of clinical informatics as a subspecialty of Pathology from 1991 through 1995, [3] but at the time it seemed that informatics was more of an art than a testable body of knowledge. Nevertheless, medical informatics was considered to be a "very young field," [4] particularly within Pathology. [5,6] Informatics has since rapidly grown [7] and has had an ever expanding impact on Pathology. [8–12] With a growing membership in API, and consistently strong attendance figures at Pathology Informatics conferences, it is the perception of the API Governing Council that there is a sufficient author and an audience base for a high-quality Pathology Informatics-focused journal. This journal aims to publish broadly on all aspects related to Pathology Informatics and to freely disseminate articles worldwide. JPI should be of interest not only to pathologists, but also to all laboratorians, informaticians, clinical informaticists, health IT specialists, information officers, researchers, vendors, and anyone else with an interest in informatics.

Previously, a journal called *Informatics in Pathology*(published by Grune and Stratton from around 1984-1987) existed, in the limited distribution print format of the day. Sadly, although many people in the field at the time agreed that there was a definite need for a Pathology Informatics journal, this publication ended because of an insufficient supply of proffered papers. [13] Many individuals in the field at the time were “practicing an art that was difficult to present in a traditional scientific paper” [Ray Aller, personal communication]. Today, “open-access” literature is digital, online, free of charge for the reader, and largely free of most copyright and licensing restrictions. Moreover, there is considerably better understanding of the scientific underpinnings of the practice of Pathology Informatics. With freedom of the constraints imposed by traditional, “old-school” journals, the API has wholeheartedly supported launching the JPI in an open-access format. While several other medical informatics journals are published, they do not focus primarily on issues relevant to Pathology. On the other hand, while there are other Pathology journals available for authors and readers, these journals publish only occasional informatics-related articles. Moreover, trying to write up something on informatics (e.g. lessons learned from a successful information system implementation) for one of these Pathology journals is like trying to *fit a square peg into a round hole*!

Therefore, we are pleased to offer this new vehicle for authors and readers alike who share an interest in Pathology Informatics. The scope of the journal is intended to be broad and all-inclusive, covering clinical (Anatomical Pathology and Clinical Pathology/Laboratory Medicine), research, education, and strategic topic areas. JPI will publish original research articles, technical notes, reviews, viewpoints, commentaries, editorials, book reviews, proceedings, correspondence to editors, and so forth. All submissions will be subject to peer review after scrutiny by the well-regarded editorial board that has been assembled, including expert referees in appropriate specialties. While much has been accomplished in the field of Pathology Informatics, it remains a relatively “young” and evolving discipline. Much ground remains to be covered including achieving true interoperability, universal adoption of standards, real-time merging of Anatomical and Clinical Pathology data, laboratory information system workflow and reporting optimization (e.g. in molecular informatics), and wider adoption of digital pathology/telepathology. [14,15] We hope that JPI will meet your needs and facilitate the evolution of Pathology Informatics as a discipline. We look forward to your ongoing support and feedback.
